# Association of Circulating Anti-HLA Donor-Specific Antibodies and Their Characteristics, including C1q-Binding Capacity, in Kidney Transplant Recipients with Long-Term Renal Graft Outcomes

**DOI:** 10.3390/jcm12041312

**Published:** 2023-02-07

**Authors:** Michal Gniewkiewicz, Katarzyna Czerwinska, Katarzyna Zielniok, Magdalena Durlik

**Affiliations:** 1 Department of Transplantation Medicine, Nephrology and Internal Diseases, Medical University of Warsaw, Nowogrodzka 59, 02-006 Warsaw, Poland; 2 Department of Clinical Immunology, Medical University of Warsaw, Nowogrodzka 59, 02-006 Warsaw, Poland

**Keywords:** antihuman leukocyte antigen donor-specific antibodies, C1q-binding DSA, kidney transplantation

## Abstract

Post-transplant antihuman leukocyte antigen donor-specific antibodies (anti-HLA DSAs) monitoring in kidney transplant recipients remains unclear and is currently under investigation. The pathogenicity of anti-HLA DSAs is determined by antibody classes, specificity, mean fluorescent intensity (MFI), C1q-binding capacity, and IgG subclasses. The aim of this study was to investigate the association of circulating DSAs and their characteristics with renal allograft long-term outcomes. The study included 108 consecutive patients from our transplant center who underwent kidney allograft biopsy between November 2018 and November 2020, 3 to 24 months after kidney transplantation. At the time of biopsy, patients’ sera were collected for analysis of anti-HLA DSAs. Patients were followed for a median time of 39.0 months (Q1–Q3, 29.8–45.0). Detection of anti-HLA DSAs at the time of biopsy (HR = 5.133, 95% CI 2.150–12.253, *p* = 0.0002) and their C1q-binding capacity (HR = 14.639, 95% CI 5.320–40.283, *p* ≤ 0.0001) were independent predictors of the composite of sustained 30% reduction from estimated glomerular filtration rate or death-censored graft failure. Identification of anti-HLA DSAs and their C1q-binding capacity could be useful in identifying kidney transplant recipients at risk for inferior renal allograft function and graft failure. Analysis of C1q is noninvasive, accessible, and should be considered in clinical practice in post-transplant monitoring.

## 1. Introduction

Kidney transplantation (KTx) is the treatment of choice for patients with end-stage kidney disease (ESKD) [1]. Despite improvements in short-term outcomes in kidney transplant recipients due to potent immunosuppressive therapy, advanced surgical techniques, and better post-transplant care, long-term outcomes have not improved to a similar extent [2].

Antibody-mediated rejection (ABMR) is the main cause of kidney allograft dysfunction and kidney allograft loss [3]. The presence of donor-specific antibodies (DSAs), particularly those against human leukocyte antigen (HLA), is a proven risk factor for the development of ABMR [4]. The role of nonanti-HLA DSA such as antibodies against angiotensin II type 1 receptor or against endothelin-1 type A receptor has been broadly analyzed [5,6]. Anti-HLA DSAs can be preexisting (preformed) or may develop de novo after transplantation [7]. Preformed DSAs are caused by exposure to the alloantigens during pregnancy, blood transfusion, or previous transplantation [8,9]. The virtual crossmatch is used to aid in renal allograft allocation and to avoid matching donors to recipients with preformed DSAs [10,11].

De novo anti-HLA DSAs develop after KTx in 13–27% of previously nonsensitized patients [12]. Usually, they emerge within 1-year post-transplant and are directed against HLA class II [13]. DSA screening in recipients with stable renal allograft function remains unclear and is currently under investigation [14,15]. Moreover, there is no consensus regarding the management of renal transplant recipients without allograft dysfunction with circulating de novo DSAs [16]. The impact of de novo anti-HLA DSAs on the development of ABMR is under investigation, as not all DSA-positive patients develop ABMR [17,18].

The pathogenicity of DSAs is determined by several characteristics, including antibody classes, specificity, strength (expressed by mean fluorescent intensity (MFI)), C1q-binding capacity, and IgG subclasses [19]. Antibodies against HLA class II occur more frequently than against HLA class I [20,21]. Patients with both anti-HLA DSA class I and II, or even class alone II, are at increased risk for ABMR [22]. The association between high MFI levels of DSAs and the increased occurrence of ABMR and decreased graft survival has been reported [23,24]. It has been demonstrated that C1q-binding capacity is a predictor of ABMR and correlates with graft survival [25,26]. However, it is not clear whether this increased risk is connected to complement-binding capacity or high MFI levels, as there is a strong correlation between the ability of DSAs to bind C1q and their strength [27,28]. IgG1 and IgG3 subclasses are strong complement-fixing antibodies, whereas IgG2 and IgG4 subclasses are considered noncomplement-fixing [29]. It was found that IgG3 and IgG4 are highly associated with ABMR and correlated with its phenotypes (IgG3 with acute ABMR, IgG4 with subclinical ABMR). Furthermore, IgG3 immunodominant DSAs are strongly and independently associated with allograft failure [19].

The aim of this study was to investigate, in a cohort of kidney transplant recipients from our center, the association of circulating DSAs and their characteristics, including MFI level, C1q-binding capacity, and IgG subclasses, with renal allograft function and long-term outcomes.

## 2. Materials and Methods

### 2.1. Study Design

The study included 108 consecutive patients from our transplant center (Department of Medical Transplantation, Nephrology and Internal Medicine, Medical University of Warsaw) who underwent kidney allograft biopsy between November 2018 and November 2020, 3 to 24 months after kidney transplantation from brain-dead deceased donors.

All kidney transplants required ABO blood group compatibility and a negative complement-dependent cytotoxicity crossmatch. All patients were of white ethnicity and had triple maintenance immunosuppression consisting of tacrolimus or cyclosporine, mycophenolate mofetil, and prednisone. The biopsy was performed using an 18-gauge needle with ultrasound guidance. The biopsy specimens were evaluated based on Banff criteria [30]. From all the patients at the time of biopsy, sera were collected for analysis of circulating anti-HLA DSAs and their characteristics (specificity, HLA class, MFI level, C1q-binding capacity, and IgG subclasses).

The primary outcome was the composite of sustained 30% reduction (defined as two consecutive results at least 3 months apart) from estimated glomerular filtration rate (eGFR) at biopsy or death-censored graft failure (defined as return to dialysis or retransplantation). EGFR was calculated using the Chronic Kidney Disease Epidemiology Collaboration 2009 (CKD-EPI) creatinine equation. All participants were followed through November 2022 (unless patient lost graft or died). Clinical data were obtained from the medical records.

The study was conducted in accordance with the Declaration of Helsinki and was approved by the medical ethics committee of the Medical University of Warsaw (Warsaw, Poland). Informed written consent was obtained from all patients.

### 2.2. Detection of Anti-HLA DSAs and Characterization

Sera samples were collected at the time of biopsy and stored at −80 °C for further analysis. All samples were analyzed for anti-HLA using LABScreen Mixed Class I and II (#LSM12, One Lambda, Inc., Los Angeles, CA, USA). When positive, samples were tested using LABScreen Single Antigen HLA class I and II assays (#LS1A04 and #LS2A01, One Lambda, Inc., CA, USA) on a Luminex platform. Antibodies with MFI > 500 were considered positive. Donor specificity for anti-HLA antibodies was determined by comparison of the HLA antibody specificities with the HLA of the donor for HLA-A, -B, and -DR.

The presence of C1q-binding anti-HLA DSAs was assessed using single antigen flow bead assays according to the manufacturer’s instructions (C1qScreen, #C1Q, One Lambda, Inc., CA, USA).

Detailed analysis of HLA DSA antibody subclasses was performed based on the method published by Hönger et al. [31]. The modification of the standard single antigen flow bead assay involved replacing the reporter PE-conjugated goat antihuman IgG antibody (#LS-AB2, One Lambda, Inc., CA, USA) with one of the monoclonal antibodies specific for the IgG subclass, that is, IgG1 (# mouse antihuman IgG 1 Fc-PE clone HP6001, # 9054-09), IgG2 (mouse antihuman IgG 2 Fc-PE clone 31-7-4, #9060-09), IgG3 (mouse antihuman IgG 3 Hinge-PE clone HP6050, #9210-09), and IgG4 (mouse antihuman IgG 4 Fc-PE clone HP6025, #9200-09) (all Southern Biotech, Birmingham, AL, USA). The detection of IgG subclasses 1-4 was conducted separately for each subclass on the sera of patients positive for HLA class I using the magnetic beads of the LABScreen Single Antigen HLA class I assay kit (Lot 013, #LS1A04, One Lambda, Inc., CA, USA) and the sera of patients positive for HLA class II using the magnetic beads of the LABScreen Single Antigen HLA class II assay kit (Lot 015, #LS2A01, One Lambda, Inc., CA, USA). Directly before analysis, the sera were centrifuged at 10,000× *g* for 10 min to remove aggregates or contamination. The initial procedure was conducted according to the manufacturer’s protocol. A 30 min incubation was performed on 20 μL of patient serum with 5 μL of LABScreen beads in V-bottom 96-well microplates. After washing the plate three times, in the modified part of the procedure, the beads were incubated for 30 min with 100 μL of PE-conjugated secondary antibody solution detecting IgG1, IgG2, IgG3, or IgG 4. After incubation, the beads were washed three times, resuspended in 80 μL of sterile phosphate buffered saline, and immediately proceeded to data acquisition on a Luminex LABScan 100 analyzer. The anti-HLA reactivity of the patients’ sera, corrected for nonspecific binding of the beads to negative control serum (#LS-NC, One Lambda, Inc., CA, USA), was calculated from the raw MFI values for each HLA-coated bead using HLA Fusion software v. 4.6 (#FUSPGR, One Lambda, Inc., CA, USA).

### 2.3. Statistical Analysis

The R software was used for statistical analysis. Categorical data were described as numbers (percentages), and continuous data were expressed as mean values with standard deviations or medians with quartiles 1 and 3 (Q1–Q3). The χ^2^ test or Fisher exact test was used for categorical variables, and the 2-sample t-test or Mann–Whitney test for continuous variables. The normality of distribution was assessed by the Shapiro–Wilk test. The primary study outcome was composite of sustained 30% reduction from eGFR at biopsy or death-censored graft failure. Event-free survival was estimated with the Kaplan–Meier method and compared according to anti-HLA antibody status with the use of the log-rank test. Cox proportional hazards models were used to estimate hazard ratios and 95% confidence intervals for the study outcomes. Variables with significant contributions in univariate Cox models were entered into the multiple adjusted Cox models to determine the independent association with outcomes. Backward stepwise elimination method with a *p*-value of <0.05 necessary for retention in the multivariate model was performed. *p* values of <0.05 were considered significant.

## 3. Results

### 3.1. Characteristics of Study Population

In the study, 108 consecutive kidney transplant recipients from brain-dead deceased donors who underwent kidney allograft biopsy 3 to 24 months after transplantation were included. At the time of biopsy, patients with present circulating DSAs were identified (N = 19). The characteristics of the study population at the time of biopsy are summarized in Table 1. There was no statistical difference between DSA (−) and DSA (+) patients regarding age at biopsy, sex, BMI, renal replacement therapy, cause of end-stage renal disease, cold ischemia time, HLA mismatches, and clinical characteristics, including type of calcineurin inhibitor (tacrolimus versus cyclosporine), eGFR at biopsy, proteinuria at biopsy, protocol biopsy, time from transplantation to biopsy, and occurrence of C4d deposition in biopsy. A higher percentage of patients with DSAs at the time of biopsy compared with DSA (−) patients had received a prior transplant (78.9% vs. 4.4%, *p* < 0.0001), had PRA > 5% (36.8% vs. 9.0%, *p* = 0.0048), had induction therapy (73.7% vs. 21.3%, *p* < 0.0001), had ABMR diagnosed at the time of biopsy (21.0% vs. 5.6%, *p* = 0.0492), and had anti-HLA DSAs before transplantation (47.4% vs. 10.1%, *p* = 0.0005). Out of 18 patients with anti-HLA DSAs before transplantation, 9 patients had them detected post-transplant at the time of biopsy. The characteristics of anti-HLA DSAs before transplantation were not statistically different between groups regarding number, class, and MFI of immunodominant anti-HLA DSAs.

### 3.2. Characteristics of Anti-HLA DSAs at the Time of Biopsy

Characteristics of anti-HLA DSAs at the time of biopsy are depicted in Table 2. Out of 19 patients with identified circulated anti-HLA DSAs at the time of biopsy, 7 patients (36.8%) had anti-HLA DSA class I, 10 patients (52.6%) had anti-HLA DSA class II, and 2 patients (10.5%) had anti-HLA DSA class I and II. The immunodominant anti-HLA DSAs were class I in 8 patients (42.1%) and class II in 11 patients (57.9%), with a median MFI (Q1–Q3) of 2900 (1284–4648). A total of 10 patients (52.6%) had immunodominant anti-HLA DSAs with C1q-banding capacity (3 patients with immunodominant anti-HLA DSA class I and 7 patients with immunodominant anti-HLA DSA class II). The most frequent IgG subclass of immunodominant anti-HLA DSA was IgG1 (14 patients, 73.7%), followed by IgG3 (7 patients, 36.8%), IgG4 (4 patients, 21.1%), and IgG2 (2 patients, 10.5%). The most predominant pattern was IgG1 alone (7 patients, 36.8%), followed by IgG3 alone (4 patients, 21.1%), IgG1 + IgG3 (3 patients, 15.8%), IgG1 + IgG4 (3 patients, 15.8%), IgG1 + IgG2 (1 patient, 5.3%), and IgG2 + IgG4 (1 patient, 5.3%).

### 3.3. Clinical Outcomes

Patients were followed for a median time of 39.0 months (Q1–Q3, 29.8–45.0) following the biopsy after transplantation. Clinical outcomes during follow-up are shown in Table 3. There was a statistical difference between DSA (−) and DSA (+) patients regarding eGFR at the end of follow-up (51.5 mL/min/1.73 m^2^ (Q1–Q3, 34.8–63.3) vs. 36.0 mL/min/1.73 m^2^ (Q1–Q3, 26.0–42.0), *p* = 0.0049), the occurrence of >30% decline in eGFR (10.1% vs. 57.9%, *p* < 0.0001), and death-censored graft loss (1.1% vs. 15.8%, *p* = 0.0168). There was no statistical difference regarding proteinuria at the end of follow-up. The combined endpoint was reached by 10 patients (11.2%) in the DSA (−) group vs. 11 patients (57.9%) in the DSA (+) group, *p* < 0.0001. The mean time from biopsy to the combined endpoint was 25.0 ± 10.69 months. During follow-up, four patients experienced a death-censored graft loss: one patient in the DSA (−) group and three patients in the DSA (+) group. The causes were ABMR (N = 2), BK virus nephropathy (N = 1), and recurrence of native kidney disease (N = 1). During follow-up, six patients died, of which four patients died with a functioning graft. The deaths were the result of cardiovascular causes (N = 3), infections (N = 2), and malignancy (N = 1).

### 3.4. Survival Analysis

**Figure 1:**
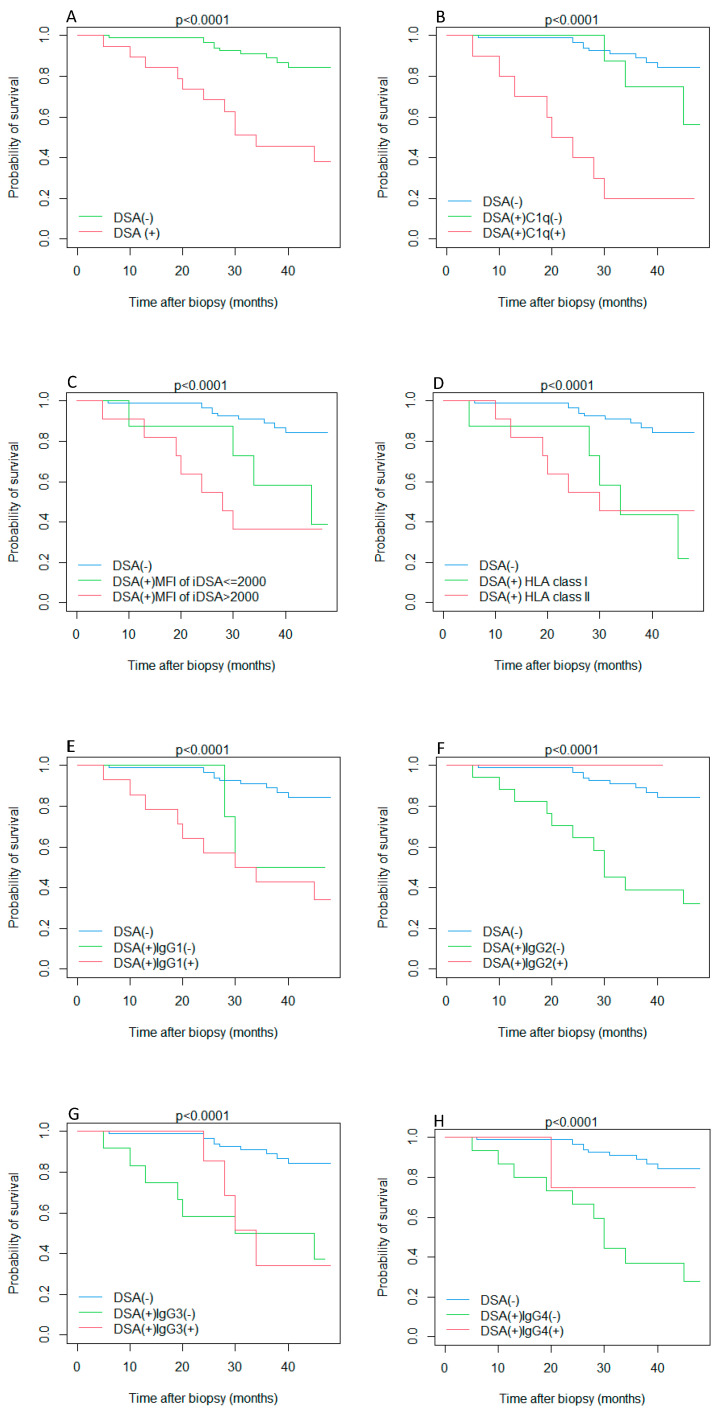
Event-free Kaplan–Meier survival curves according to detection of anti-HLA DSAs at the time of biopsy (**A**) and their characteristics: C1q- binding capacity (**B**), MFI (**C**), HLA class (**D**), IgG subclasses (**E**–**H**). Event defined as composite of sustained 30% reduction from eGFR at biopsy or death-censored graft failure. Abbreviations: DSA, donor-specific antibodies; MFI, mean fluorescent intensity; iDSA, immunodominant donor-specific antibody; HLA, human leukocyte antigen.

Combined endpoint-free survival analysis according to anti-HLA DSA status at the time of biopsy is depicted in Figure 1. Reduced survival was associated with the presence of circulating DSAs (*p* < 0.0001) and C1q-banding capacity (DSA (+) C1q (−) vs. DSA (+) C1q (+), *p*-value = 0.01). No statistical difference was observed between DSA (+) Kaplan–Meier curves regarding MFI > 2000 vs. MFI ≤ 2000 (*p* = 0.4), HLA class I vs. HLA class II (*p* = 0.9), IgG1 (+) vs. IgG1 (−) (*p* = 0.5), IgG2 (+) vs. IgG2 (−) (*p* = 0.2), IgG3 (+) vs. IgG3 (−) (*p* = 0.8), and IgG4 (+) vs. IgG4 (−) (*p* = 0.2).

Univariate and multivariate Cox regression models for risk of the combined endpoint of >30% decline in eGFR or graft loss is shown in Table 4. The following independent predictors of >30% decline in eGFR or graft loss were identified in all models: age of the donor and proteinuria at the time of biopsy ≥50 mg/dl. Occurrence of circulating anti-HLA DSAs at the time of biopsy (HR = 5.133, 95% CI 2.150–12.253, *p* = 0.0002) and C1q-binding capacity of anti-HLA DSAs at the time of biopsy (HR = 14.639, 95% CI 5.320–40.283, *p* < 0.0001) were independent predictors of the >30% decline in eGFR or graft loss, and each variable was analyzed separately in a correspondent multivariate model.

## 4. Discussion

In this study, the utility of the identification of anti-HLAs and their characteristics after kidney transplantation to determine patients at risk for inferior allograft function and allograft failure was analyzed. A total of 19 out of 108 patients (17.6%) were identified with circulating anti-HLA DSAs at the time of biopsy, 3 to 24 months after kidney transplantation. Ten patients (52.6%) had immunodominant anti-HLA DSAs with C1q-banding capacity. The most frequent IgG subclass of immunodominant anti-HLA DSA was IgG1, followed by IgG3. These results are consistent with the available literature [31,32]. The development of anti-HLA DSAs may be caused before transplantation by pregnancy, blood transfusion, or prior transplantation, or anti-HLA DSAs may develop after transplantation [8]. In patients with circulating anti-HLA DSAs, 47.4% had anti-HLA DSAs detected before transplantation, and 78.9% had previous transplantation. There are several clinical events that contribute to the formation of de novo anti-HLA DSAs, including blood transfusion, pregnancy, homograft implantation, nonadherence, and immunosuppression minimization [33,34,35]. Patients with de novo anti-HLA DSAs display a 25% to 53% incidence of subclinical ABMR at the time of anti-HLA DSA identification, whereas 31% to 50% of patients with preformed anti-HLA DSAs develop subclinical ABMR by 3 months post-transplant [36]. Patients with anti-HLA DSAs have an increased risk of chronic ABMR and kidney allograft loss compared with patients with preformed anti-HLA DSAs [37,38]. However, the detection of circulating anti-HLA DSAs, regardless of their status, is associated with increased expression of rejection transcripts in renal transplant biopsies classified as no rejection [39].

The identification of anti-HLA DSAs was an independent predictor of the composite of sustained 30% reduction from eGFR at biopsy or death-censored graft failure. Previous studies showed an association between circulating anti-HLA DSAs and increased C4d deposition in the peritubular capillaries of the allograft, as well as increased microvascular inflammation, which leads to ABMR and kidney graft failure [40,41,42]. Moreover, it was discovered that the course of fibrogenesis in renal allograft is significantly accelerated by circulating anti-HLA DSAs independent of ABMR [43]. Recently, the strong association of proinflammatory blood cytokine profiles was demonstrated with the presence of HLA DSAs, even in the absence of histology of rejection [44]. In previous papers, it was reported that the presence of anti-HLA DSAs was associated with inferior graft outcomes and graft failure [45,46]. However, some studies demonstrated contrasting findings [47].

To better evaluate the predictive value of anti-HLA DSAs for renal graft outcomes, their characteristics, including MFI level, C1q-binding capacity, and IgG subclasses, have been broadly analyzed [19]. In this study, the C1q-binding capacity of anti-HLA DSAs was proved to be an independent predictor of inferior renal graft outcomes. This is consistent with what has been found in previous papers [25,26]. A recent meta-analysis by Kang et al., which included relevant studies comparing the clinical outcomes between DSA C1q (+) and DSA C1q (−) kidney transplant recipients, demonstrated that C1q-binding DSAs are associated with increased risks of ABMR, renal allograft failure, and patient death [48]. The pathogenic potential of C1q DSA is explained by the fact that binding complement fraction C1 is the first step in the activation of the classic complement pathway, which results in the formation of a membrane attack complex that contributes to vascular injury and causes target cell damage [49]. Detection of C1q DSA could have therapeutic consequences, as there are available complement-targeting agents, such as eculizumab [26,50]. It has been shown that circulating C1q-binding anti-HLA DSAs after therapeutic intervention for ABMR may reflect the effect of treatment and predict long-term allograft survival [51]. In a recent study, it was demonstrated that timely treatment with an augmented immunosuppressive protocol of C1q-binding anti-HLA DSA-associated ABMR, with early detection and elimination of C1q-binding anti-HLAs, may be associated with better outcomes [52]. A large multicenter randomized clinical trial is needed to assess the role of the C1q-binding capacity of anti-HLA DSAs in kidney transplant recipients.

The study included a relatively small number of patients with DSA positivity; therefore, it was not possible to fully characterize the utility of IgG subclasses on renal allograft outcomes. IgG subclass assays are not currently commercially available and are not used in clinical practice. In line with previous studies, the IgG1 subclass was the most common [53]. Moreover, it is worth discussing the fact that IgG2 and IgG4 subclasses were identified but only in combination with other subclasses. A similar pattern of results suggesting the evolution of an immune response was obtained by Ponsirenas et al. [54]. It was hypothesized that human B cells follow a programmed sequence of immunoglobulin class switching from IgM to IgG3, then to IgG1 and to IgG2 and, finally, to IgG4 [55]. That could be supported by the finding that IgG2 and IgG4 subclasses of anti-HLA DSAs were detected in eluates of rejected renal allografts [56].

In our study population, patients with immunodominant anti-HLA DSA MFI values of >2000 vs. ≤2000 did not have significantly different event-free survival. Analysis of MFI values is challenging, as there is no international threshold for MFI values and a lack of standardization [57]. Therefore, regulatory agencies do not accept single antigen bead assays as quantitative measurements [51].

The results of the study must be interpreted with caution, and several limitations should be borne in mind. This was a single-center study with a relatively small number of patients with DSA positivity. The presence of DSA in patients was tested at different points in time but within 3 to 24 months after kidney transplantation at the time of renal allograft biopsy. The DSAs and their characteristics were not monitored during follow-up. The DSAs against HLA-C, -DP, and -DQ are not included, as HLA-C, -DP, and -DQ typing were not available for kidney donors. The heterogeneity in the induction of immunosuppression treatment could not be avoided.

To conclude, our data reported that the identification of circulating anti-HLA DSAs and their C1q-binding capacity are independent predictors for inferior renal allograft function and graft failure after kidney transplantation. Even though analysis of C1q is limited by its cost, it is noninvasive, easily accessible, and should be considered in routine clinical practice in post-transplant monitoring. This could improve the optimization of post-transplant care. Our research adds to the literature supporting the use of the C1q assay in the immunological stratification of kidney transplant recipients for long-term renal allograft outcomes.

## Figures and Tables

**Table 1:** Characteristics of the study population.

	All Patients (N = 108)	DSA (−) (N = 89)	DSA (+) (N = 19)	*p*-Value
**Recipient characteristic**				
Age at biopsy, years, median (Q1–Q3)	48.5 (38.8–61.0)	46.0 (38.0–61.0)	54.0 (44.0–58.5)	0.6054
Male, n (%)	69 (63.9%)	58 (65.2%)	11 (57.9%)	0.7368
Body mass index at biopsy, kg/m^2^, mean ± SD	25.24 ± 3.848	25.52 ± 3.934	23.93 ± 3.188	0.1031
Previous transplantation, n (%)	19 (17.6%)	4 (4.4%)	15 (78.9%)	<0.0001
Renal replacement therapy, n (%)				0.6326
Pre-emptive transplantation	12 (11.1%)	11 (12.4%)	1 (5.3%)	
Hemodialysis	83 (76.9%)	67 (75.2%)	16 (84.1%)	
Peritoneal dialysis	13 (12.0%)	11 (12.4%)	2 (10.6%)	
Cause of ESRD, n (%)				0.2384
Glomerulonephritis	48 (44.4%)	36 (40.4%)	12 (63.2%)	
ADPKD	19 (17.6%)	15 (16.8%)	4 (21.1%)	
Diabetes	16 (14.8%)	14 (15.7%)	2 (10.5%)	
Congenital anomaly	5 (4.6%)	5 (5.6%)	0	
Other	20 (18.5%)	19 (21.5%)	1 (5.2%)	
Diabetes, n (%)	31 (28.7%)	26 (29.2%)	5 (26.3%)	
**Donor characteristic**				
Age, years, mean ± SD	46.2 ± 14.88	45.0 ± 14.9	52.0 ± 13.8	0.0616
Male, n (%)	68 (63.0%)	58 (65.2%)	10 (52.6%)	0.4439
**Transplant characteristic**				
Cold ischemia time, minutes, mean ± SD	1268 ± 575.4	1239 ± 582.9	1416 ± 559.8	0.4972
Induction therapy, n (%)				<0.0001
None	75 (69.4%)	70 (78.7%)	5 (26.3%)	
Basiliximab	18 (16.7%)	10 (11.2%)	8 (42.1%)	
ATG	15 (13.9%)	9 (10.1%)	6 (31.6%)	
HLA mismatches, median, (Q1–Q3)				
A	1.0 (1.0–2.0)	1.0 (1.0–2.0)	1.0 (1.0–1.5)	0.5127
B	1.0 (1.0–2.0)	1.0 (1.0–2.0)	1.0 (1.0–2.0)	0.4485
DR	1.0 (1.0–1.0)	1.0 (1.0–1.0)	1.0 (1.0–1.0)	0.1550
Total	3.0 (3.0–4.0)	3.0 (3.0–4.0)	4.0 (3.0–4.0)	0.4000
Panel-reactive antibody >5%, n (%)	15 (13.9%)	8 (9.0%)	7 (36.8%)	0.0048
Panel-reactive antibody, median (Q1–Q3) [min–max]	0 (0–0) [0–86]	0 (0–0) [0–86]	0 (0–18.5) [0–66]	0.0043
**Clinical characteristic**				
Immunosuppression, n (%)				0.3223
Tacrolimus	106 (98.1%)	88 (89.9%)	18 (94.7%)	
Cyclosporine	2 (1.9%)	1 (1.1%)	1 (5.3%)	
eGFR at biopsy, mL/min/1.73 m^2^, mean ± SD	53.2 ± 20.76	54.6 ± 21.24	46.3 ± 17.19	0.1107
Proteinuria at biopsy ≥ 50 mg/dL, n (%)	16 (14.8%)	11 (12.6%)	5 (26.3%)	0.2306
Proteinuria at biopsy, median (Q1–Q3) [min–max]	0 (0–10.0) [0–100]	0 (0–10.0) [0–100]	0 (0–30.0) [0–50]	0.1363
Protocol biopsy n (%)	61 (56.5%)	52 (58.4%)	9 (47.4%)	0.4483
Time from transplantation to biopsy, months, median (Q1–Q3)	5.0 (3.0–12.0)	5.0 (3.0–12.0)	4.0 (3.0–10.5)	0.4374
ABMR at the time of biopsy, n (%)	9 (8.3%)	5 (5.6%)	4 (21.0%)	0.0492
C4d in biopsy, n (%)	14 (13.0%)	10 (11.2%)	4 (21.1%)	0.2651
Anti-HLA DSA before transplantation, n (%)	18 (16.7%)	9 (10.1%)	9 (47.4%)	0.0005
Number, median (Q1–Q3)	1.0 (1.0–1.8)	1.0 (1.0–1.0)	1.0 (1.0–2.0)	0.5008
Anti-HLA DSA class, n				1
I	11	6	5	
II	6	3	3	
I + II	1	0	1	
MFI of immunodominant anti-HLA DSA before transplantation, mean (SD)	2009 ± 1230.5	1807 ± 1347.3	2211 ± 1144.6	0.5022

Abbreviations: DSA, donor-specific antibodies; Q1-Q3, quartile 1–3; SD, standard deviation; ESRD, end-stage renal disease; ADPKD, autosomal dominant polycystic kidney disease; ATG, anti-thymocyte globulin; HLA, human leukocyte antigen; eGFR, estimated glomerular filtration rate; ABMR, antibody-mediated rejection; MFI, mean fluorescent intensity.

**Table 2:** Characteristics of anti-HLA DSAs.

All Anti-HLA DSA	
Number, median (Q1–Q3)	1.0 (1.0–1.0)
HLA class specificity, n (%)	
I	7 (36.8)
II	10 (52.6)
I + II	2 (10.5)
**iDSA**	
HLA class specificity, n (%)	
I	8 (42.1)
II	11 (57.9)
MFI, median (Q1–Q3)	2900 (1284–4648)
C1q binding, n (%)	10 (52.6)
IgG subclasses, n (%)	
IgG1	14 (73.7)
IgG2	2 (10.5)
IgG3	7 (36.8)
IgG4	4 (21.1)

Abbreviations: HLA, human leukocyte antigen; DSA, donor-specific antibodies; Q1–Q3, quartile 1–3; iDSA, immunodominant specific antibody; MFI, mean fluorescent intensity.

**Table 3:** Clinical outcomes.

	All Patients (N = 108)	DSA (−) (N = 89)	DSA (+) (N = 19)	*p*-Value
Follow-up postbiopsy, months, median (Q1–Q3)	39.0 (29.8–45.0)	37.0 (28.0–44.0)	44.0 (37.5–47.0)	0.0631
eGFR at the end of follow-up, mL/min/1.73 m^2^, median (Q1–Q3)	48.0 (33.0–62.0)	51.5 (34.8–63.3)	36.0 (26.0–42.0)	0.0049
Proteinuria at the end of follow-up, ≥50 mg/dL, n (%)	6 (5.6)	3 (3.4)	3 (15.8)	0.1110
Proteinuria at the end of follow-up, median (Q1–Q3) [min–max]	0 (0–0) [0–311.0]	0 (0–0) [0–311.0]	0 (0–6.8) [0–81.0]	0.4113
>30% decline in eGFR, n (%)	20 (18.5)	9 (10.1)	11 (57.9)	<0.0001
Death-censored graft loss, n (%)	4 (3.7)	1 (1.1)	3 (15.8)	0.0168
Combined endpoint, >30% decline in eGFR or graft loss, n (%)	21 (19.4)	10 (11.2)	11 (57.9)	<0.0001
Time from biopsy to combined endpoint, months, mean ± SD	25.0 ± 10.69	26.9 ± 9.79	23.4 ± 11.60	0.482
Death event, n (%)	6 (5.6)	4 (4.4)	2 (10.5)	0.2842

Abbreviations: DSA, donor-specific antibodies; Q1-Q3, quartile 1–3; eGFR, estimated glomerular filtration rate; SD, standard deviation.

**Table 4:** Univariate and multivariate Cox regression models for risk of the combined endpoint of >30% decline in eGFR or death-censored graft loss.

	Univariate			Multivariate 1			Multivariate 2		
	HR	95% CI	*p*-Value	HR	95% CI	*p*-Value	HR	95% CI	*p*-Value
**Recipient characteristic**									
Age at biopsy (per 1-year increase)	1.001	0.969–1.035	0.9486						
Male (vs. female)	1.291	0.518–3.216	0.5835						
Body mass index at biopsy (per 1 kg/m^2^ increase)	0.953	0.855–1.063	0.3918						
Previous transplantation (yes vs. no)	3.728	1.568–8.864	0.0029						
Renal replacement therapy									
Pre-emptive	Ref.								
Hemodialysis	0.821	0.239–2.822	0.754						
Peritoneal dialysis	0.761	0.127–4.571	0.765						
**Donor characteristic**									
Age (per 1-year increase)	1.045	1.010–1.081	0.0106	1.034	1.002–1.067	0.0382	1.040	1.005–1.077	0.0259
Male (vs. female)	0.353	0.146–0.852	0.0205						
**Transplant characteristic**									
Induction therapy									
No	Ref.								
Basiliximab	3.294	1.224–8.862	0.0182						
ATG	2.925	0.980–8.735	0.0545						
Total HLA mismatches (per 1-mismatch increase)	1.349	0.860–2.116	0.1925						
Panel-reactive antibody >5% (vs. ≤5%)	1.309	0.479–3.579	0.5993						
**Clinical characteristic**									
eGFR at biopsy (per 1-mL/min/1.73 m^2^ increase)	0.992	0.971–1.013	0.4490						
Proteinuria at biopsy ≥50 mg/dL (vs. <50 mg/dL)	2.628	1.019–6.776	0.0457	2.612	1.006–6.783	0.0486	3.504	1.281–9.588	0.0146
ABMR at the time of biopsy (yes vs. no)	2.361	0.692–8.054	0.1698						
C4d in biopsy (yes vs. no)	1.426	0.479–4.245	0.5234						
Anti-HLA DSAs before transplantation (yes vs. no)	3.031	1.221–7.524	0.0168						
Anti-HLA DSAs at biopsy (yes vs. no) *	6.01	2.546–14.190	<0.0001	5.133	2.150–12.253	0.0002			
C1q-binding status									
No anti-HLA DSAs at biopsy **	Ref.						Ref.		
Anti-HLA DSAs (+) C1q (−) at biopsy **	2.501	0.687–9.107	0.164				1.927	0.524–7.084	0.3236
Anti-HLA DSAs (+) C1q (+) at biopsy **	12.844	5.010–32.926	<0.0001				14.639	5.320–40.283	<0.0001

* Included in multivariate model 1. ** Included in multivariate model 2. Abbreviations: HR, hazard ratio; CI, confidence interval; ATG, antithymocyte globulin; HLA, human leukocyte antigen; eGFR, estimated glomerular filtration rate; ABMR, antibody-mediated rejection; DSA, donor-specific antibodies.

## Data Availability

The data presented in this study are available on request from the corresponding author.

## References

[1] McCormick F., Held P.J., Chertow G.M. (2018). The Terrible Toll of the Kidney Shortage. J. Am. Soc. Nephrol..

[2] Coemans M., Süsal C., Döhler B., Anglicheau D., Giral M., Bestard O., Legendre C., Emonds M.P., Kuypers D., Molenberghs G. (2018). Analyses of the short- and long-term graft survival after kidney transplantation in Europe between 1986 and 2015. Kidney Int..

[3] Sellarés J., de Freitas D.G., Mengel M., Reeve J., Einecke G., Sis B., Hidalgo L.G., Famulski K., Matas A., Halloran P.F. (2012). Understanding the causes of kidney transplant failure: The dominant role of antibody-mediated rejection and nonadherence. Am. J. Transplant..

[4] Cioni M., Nocera A., Innocente A., Tagliamacco A., Trivelli A., Basso S., Quartuccio G., Fontana I., Magnasco A., Drago F. (2017). De Novo Donor-Specific HLA Antibodies Developing Early or Late after Transplant Are Associated with the Same Risk of Graft Damage and Loss in Nonsensitized Kidney Recipients. J. Immunol. Res..

[5] El Band J.E.K., Llorente S., Martinez-Garcia P., Alfaro R., Jimenez-Coll V., Boix F., Galián J.A., Martinez-Banaclocha H., Botella C., Moya-Quiles M.R. (2021). Evaluation of Antibodies Directed Against Two GPCRs, Anti-AT1R and Anti-ETAR, on Kidney Transplant Outcome. Curr. Protein Pept. Sci..

[6] Filippone E.J., Farber J.L. (2021). Histologic Antibody-mediated Kidney Allograft Rejection in the Absence of Donor-specific HLA Antibodies. Transplantation.

[7] Hung S.Y., Lin T.M., Chang M.Y., Wang H.H., Lee Y.C., Ho L.C., Chen Y.T., Hung C.M., Liou H.H. (2014). Risk factors of sensitization to human leukocyte antigen in end-stage renal disease patients. Hum. Immunol..

[8] Yeung M.Y. (2020). Pre-formed DSA and kidney allograft outcomes. Braz. J. Nephrol..

[9] Oweira H., Ramouz A., Ghamarnejad O., Khajeh E., Ali-Hasan-Al-Saegh S., Nikbakhsh R., Reißfelder C., Rahbari N., Mehrabi A., Sadeghi M. (2022). Risk Factors of Rejection in Renal Transplant Recipients: A Narrative Review. J. Clin. Med..

[10] Olszowska-Zaremba N., Zagożdżon R., Gozdowska J. (2022). Accuracy of virtual crossmatch (VXM) prediction of physical crossmatch (PXM) results of donor specific antibody (DSA) in routine pretransplant settings-a single-center experience. Transpl. Immunol..

[11] Leal R., Pardinhas C., Martinho A., Sá H.O., Figueiredo A., Alves R. (2022). Strategies to Overcome HLA Sensitization and Improve Access to Retransplantation after Kidney Graft Loss. J. Clin. Med..

[12] Matignon M., Pilon C., Commereuc M., Grondin C., Leibler C., Kofman T., Audard V., Cohen J., Canoui-Poitrine F., Grimbert P. (2017). Intravenous immunoglobulin therapy in kidney transplant recipients with de novo DSA: Results of an observational study. PLoS ONE.

[13] Heilman R.L., Nijim A., Desmarteau Y.M., Khamash H., Pando M.J., Smith M.L., Chakkera H.A., Huskey J., Valdez R., Reddy K.S. (2014). De novo donor-specific human leukocyte antigen antibodies early after kidney transplantation. Transplantation.

[14] Tambur A.R., Campbell P., Chong A.S., Feng S., Ford M.L., Gebel H., Gill R.G., Kelsoe G., Kosmoliaptsis V., Mannon R.B. (2020). Sensitization in transplantation: Assessment of risk (STAR) 2019 Working Group Meeting Report. Am. J. Transplant..

[15] Sharma A., Jorgensen D.R., Mehta R.B., Sood P., Puttarajappa C.M., Wu C.M., Tevar A.D., Molinari M., Zeevi A., Hariharan S. (2022). The Clinical Impact of Anti-HLA Donor Specific Antibody Detection Through First Year Screening on Stable Kidney Transplant Recipients. Transpl. Int..

[16] Yamamoto T., Watarai Y., Takeda A., Tsujita M., Hiramitsu T., Goto N., Narumi S., Katayama A., Morozumi K., Uchida K. (2016). De Novo Anti-HLA DSA Characteristics and Subclinical Antibody-Mediated Kidney Allograft Injury. Transplantation.

[17] Nakamura K., Sawada A., Kita Y., Kono J., Masui K., Sato T., Sano T., Goto T., Akamatsu S., Ogawa O. (2022). Clinical characteristics of renal transplant recipients who developed de novo donor-specific antigen in Kyoto University Hospital: A case series. Ren. Replace Ther..

[18] Wu K., Schmidt D., López Del Moral C., Osmanodja B., Lachmann N., Zhang Q., Halleck F., Choi M., Bachmann F., Ronicke S. (2021). Poor Long-Term Renal Allograft Survival in Patients with Chronic Antibody-Mediated Rejection, Irrespective of Treatment-A Single Center Retrospective Study. J. Clin. Med..

[19] Lefaucheur C., Viglietti D., Bentlejewski C., Duong van Huyen J.P., Vernerey D., Aubert O., Verine J., Jouven X., Legendre C., Glotz D. (2016). IgG Donor-Specific Anti-Human HLA Antibody Subclasses and Kidney Allograft Antibody-Mediated Injury. J. Am. Soc. Nephrol..

[20] Tatapudi V.S., Kopchaliiska D., da Gente G.J., Buenaventura O.F., Singh M., Laszik Z., Adey D.B., Rajalingam R. (2021). Solid-Phase C1q/C3d Fixing Readouts Correlate with High Median Fluorescence Intensity (MFI) De Novo Donor-Specific HLA Antibodies and C4d⁺ Antibody-Mediated Rejection in Kidney Transplant Recipients. Ann. Transplant..

[21] Butiu M., Obrisca B., Sibulesky L., Bakthavatsalam R., Smith K.D., Gimferrer I., Warner P., Ismail G., Leca N. (2022). Donor-derived Cell-free DNA Complements De Novo Class II DSA in Detecting Late Alloimmune Injury Post Kidney Transplantation. Transpl. Direct.

[22] Willicombe M., Roufosse C., Brookes P., Galliford J.W., McLean A.G., Dorling A., Warrens A.N., Cook T.H., Cairns T.D., Taube D. (2011). Antibody-mediated rejection after alemtuzumab induction: Incidence, risk factors, and predictors of poor outcome. Transplantation.

[23] Lefaucheur C., Loupy A., Hill G.S., Andrade J., Nochy D., Antoine C., Gautreau C., Charron D., Glotz D., Suberbielle-Boissel C. (2010). Preexisting donor-specific HLA antibodies predict outcome in kidney transplantation. J. Am. Soc. Nephrol..

[24] Mizutani K., Terasaki P., Hamdani E., Esquenazi V., Rosen A., Miller J., Ozawa M. (2007). The importance of anti-HLA-specific antibody strength in monitoring kidney transplant patients. Am. J. Transplant..

[25] Malheiro J., Tafulo S., Dias L., Martins S., Fonseca I., Beirão I., Castro-Henriques A., Cabrita A. (2017). Determining donor-specific antibody C1q-binding ability improves the prediction of antibody-mediated rejection in human leucocyte antigen-incompatible kidney transplantation. Transpl. Int..

[26] Loupy A., Lefaucheur C., Vernerey D., Prugger C., Duong van Huyen J.P., Mooney N., Suberbielle C., Frémeaux-Bacchi V., Méjean A., Desgrandchamps F. (2013). Complement-binding anti-HLA antibodies and kidney-allograft survival. N. Engl. J. Med..

[27] Yell M., Muth B.L., Kaufman D.B., Djamali A., Ellis T.M. (2015). C1q Binding Activity of De Novo Donor-specific HLA Antibodies in Renal Transplant Recipients With and Without Antibody-mediated Rejection. Transplantation.

[28] Navas A., Molina J., Agüera M.L., Guler I., Jurado A., Rodríguez-Benot A., Alonso C., Solana R. (2019). Characterization of the C1q-Binding Ability and the IgG1-4 Subclass Profile of Preformed Anti-HLA Antibodies by Solid-Phase Assays. Front. Immunol..

[29] Schaub S., Hönger G., Amico P. (2014). The complexity of the humoral immune response against HLA antigens. Transpl. Int..

[30] Haas M., Loupy A., Lefaucheur C., Roufosse C., Glotz D., Seron D., Nankivell B.J., Halloran P.F., Colvin R.B., Akalin E. (2018). The Banff 2017 Kidney Meeting Report: Revised diagnostic criteria for chronic active T cell-mediated rejection, antibody-mediated rejection, and prospects for integrative endpoints for next-generation clinical trials. Am. J. Transplant..

[31] Hönger G., Hopfer H., Arnold M.L., Spriewald B.M., Schaub S., Amico P. (2011). Pretransplant IgG subclasses of donor-specific human leukocyte antigen antibodies and development of antibody-mediated rejection. Transplantation.

[32] Freitas M.C., Rebellato L.M., Ozawa M., Nguyen A., Sasaki N., Everly M., Briley K.P., Haisch C.E., Bolin P., Parker K. (2013). The role of immunoglobulin-G subclasses and C1q in de novo HLA-DQ donor-specific antibody kidney transplantation outcomes. Transplantation.

[33] Snanoudj R., Kamar N., Cassuto E., Caillard S., Metzger M., Merville P., Thierry A., Jollet I., Grimbert P., Anglicheau D. (2019). Epitope load identifies kidney transplant recipients at risk of allosensitization following minimization of immunosuppression. Kidney Int..

[34] Sharma A., Cherukuri A., Mehta R.B., Sood P., Hariharan S. (2019). High Calcineurin Inhibitor Intrapatient Variability Is Associated With Renal Allograft Inflammation, Chronicity, and Graft Loss. Transplant. Direct.

[35] Coti I., Wenda S., Andreeva A., Kocher A., Laufer G., Fischer G., Andreas M. (2020). Donor-specific HLA antibodies after fresh decellularized vs. cryopreserved native allograft implantation. Hla.

[36] Lefaucheur C., Louis K., Morris A.B., Taupin J.L., Nickerson P., Tambur A.R., Gebel H.M., Reed E.F. (2023). Clinical recommendations for posttransplant assessment of anti-HLA (Human Leukocyte Antigen) donor-specific antibodies: A Sensitization in Transplantation: Assessment of Risk consensus document. Am. J. Transplant..

[37] Aubert O., Loupy A., Hidalgo L., Duong van Huyen J.-P., Higgins S., Viglietti D., Jouven X., Glotz D., Legendre C., Lefaucheur C. (2017). Antibody-Mediated Rejection Due to Preexisting versus De Novo Donor-Specific Antibodies in Kidney Allograft Recipients. J. Am. Soc. Nephrol..

[38] Haas M., Mirocha J., Reinsmoen N.L., Vo A.A., Choi J., Kahwaji J.M., Peng A., Villicana R., Jordan S.C. (2017). Differences in pathologic features and graft outcomes in antibody-mediated rejection of renal allografts due to persistent/recurrent versus de novo donor-specific antibodies. Kidney Int..

[39] Madill-Thomsen K.S., Böhmig G.A., Bromberg J., Einecke G., Eskandary F., Gupta G., Hidalgo L.G., Myslak M., Viklicky O., Perkowska-Ptasinska A. (2021). Donor-Specific Antibody Is Associated with Increased Expression of Rejection Transcripts in Renal Transplant Biopsies Classified as No Rejection. J. Am. Soc. Nephrol..

[40] Loupy A., Hill G.S., Suberbielle C., Charron D., Anglicheau D., Zuber J., Timsit M.O., Duong J.P., Bruneval P., Vernerey D. (2011). Significance of C4d Banff scores in early protocol biopsies of kidney transplant recipients with preformed donor-specific antibodies (DSA). Am. J. Transplant..

[41] Einecke G., Sis B., Reeve J., Mengel M., Campbell P.M., Hidalgo L.G., Kaplan B., Halloran P.F. (2009). Antibody-mediated microcirculation injury is the major cause of late kidney transplant failure. Am. J. Transplant..

[42] Lebraud E., Eloudzeri M., Rabant M., Lamarthée B., Anglicheau D. (2022). Microvascular Inflammation of the Renal Allograft: A Reappraisal of the Underlying Mechanisms. Front. Immunol..

[43] Gosset C., Viglietti D., Rabant M., Vérine J., Aubert O., Glotz D., Legendre C., Taupin J.L., Duong Van-Huyen J.P., Loupy A. (2017). Circulating donor-specific anti-HLA antibodies are a major factor in premature and accelerated allograft fibrosis. Kidney Int..

[44] Van Loon E., Lamarthée B., Barba T., Claes S., Coemans M., de Loor H., Emonds M.P., Koshy P., Kuypers D., Proost P. (2022). Circulating Donor-Specific Anti-HLA Antibodies Associate With Immune Activation Independent of Kidney Transplant Histopathological Findings. Front. Immunol..

[45] Mohan S., Palanisamy A., Tsapepas D., Tanriover B., Crew R.J., Dube G., Ratner L.E., Cohen D.J., Radhakrishnan J. (2012). Donor-specific antibodies adversely affect kidney allograft outcomes. J. Am. Soc. Nephrol..

[46] Terasaki P.I., Ozawa M., Castro R. (2007). Four-year follow-up of a prospective trial of HLA and MICA antibodies on kidney graft survival. Am. J. Transplant..

[47] Parajuli S., Joachim E., Alagusundaramoorthy S., Aziz F., Blazel J., Garg N., Muth B., Mohamed M., Redfield R.R., Mandelbrot D.A. (2019). Donor-Specific Antibodies in the Absence of Rejection Are Not a Risk Factor for Allograft Failure. Kidney Int. Rep..

[48] Kang Z.Y., Liu C., Liu W., Li D.H. (2022). Effect of C1q-binding donor-specific anti-HLA antibodies on the clinical outcomes of patients after renal transplantation: A systematic review and meta-analysis. Transpl. Immunol..

[49] Murata K., Baldwin W.M. (2009). Mechanisms of complement activation, C4d deposition, and their contribution to the pathogenesis of antibody-mediated rejection. Transplant. Rev..

[50] Patel J.K., Coutance G., Loupy A., Dilibero D., Hamilton M., Kittleson M., Kransdorf E., Azarbal B., Seguchi O., Zhang X. (2021). Complement inhibition for prevention of antibody-mediated rejection in immunologically high-risk heart allograft recipients. Am. J. Transplant..

[51] Viglietti D., Bouatou Y., Kheav V.D., Aubert O., Suberbielle-Boissel C., Glotz D., Legendre C., Taupin J.L., Zeevi A., Loupy A. (2018). Complement-binding anti-HLA antibodies are independent predictors of response to treatment in kidney recipients with antibody-mediated rejection. Kidney Int..

[52] Sigurjonsdottir V.K., Purington N., Chaudhuri A., Zhang B.M., Fernandez-Vina M., Palsson R., Kambham N., Charu V., Piburn K., Maestretti L. (2022). Complement-Binding Donor-Specific Anti-HLA Antibodies: Biomarker for Immunologic Risk Stratification in Pediatric Kidney Transplantation Recipients. Transpl. Int..

[53] Lowe D., Higgins R., Zehnder D., Briggs D.C. (2013). Significant IgG subclass heterogeneity in HLA-specific antibodies: Implications for pathogenicity, prognosis, and the rejection response. Hum. Immunol..

[54] Ponsirenas R.V.G., Cazarote H.B., Araújo S.A., Wanderley D.C., Shimakura S., Valdameri J.S., Contieri F.L.C., von Glehn C., Susin M.F., Sotomaior V.S. (2018). Anti-HLA Donor-Specific IgG Subclasses and C1q-binding Evolution in Posttransplant Monitoring. Transpl. Direct.

[55] Collins A.M., Jackson K.J. (2013). A Temporal Model of Human IgE and IgG Antibody Function. Front. Immunol..

[56] Heinemann F.M., Roth I., Rebmann V., Arnold M.L., Witzke O., Wilde B., Spriewald B.M., Grosse-Wilde H. (2007). Immunoglobulin isotype-specific characterization of anti-human leukocyte antigen antibodies eluted from explanted renal allografts. Hum. Immunol..

[57] Gu Y., Koh R.W.K., Lai M.L., Pochinco D., Teo R.Z.C., Chan M., Murali T.M., Liew C.W., Wong Y.H., Gascoigne N.R.J. (2020). Defining the structural basis for human leukocyte antigen reactivity in clinical transplantation. Sci. Rep..

